# Machine Learning versus Cox Models for Predicting Overall Survival in Patients with Osteosarcoma: A Retrospective Analysis of the EURAMOS-1 Clinical Trial Data

**DOI:** 10.3390/cancers16162880

**Published:** 2024-08-19

**Authors:** Marta Spreafico, Audinga-Dea Hazewinkel, Michiel A. J. van de Sande, Hans Gelderblom, Marta Fiocco

**Affiliations:** 1 Mathematical Institute, Leiden University, Einsteinweg 55, 2333 CC Leiden, The Netherlands; m.fiocco@math.leidenuniv.nl; 2 Department of Biomedical Data Sciences—Medical Statistics, Leiden University Medical Center, Albinusdreef 2, 2333 ZA Leiden, The Netherlands; 3 Department of Medical Statistics, Faculty of Epidemiology and Population Health, London School of Hygiene & Tropical Medicine, Keppel Street, London WC1E 7HT, UK; dea.hazewinkel@lshtm.ac.uk; 4 Department of Orthopedic Surgery, Leiden University Medical Center, Albinusdreef 2, 2333 ZA Leiden, The Netherlands; m.a.j.van_de_sande@lumc.nl; 5 Department of Orthopedic Surgery, Princess Máxima Center for Pediatric Oncology, Heidelberglaan 25, 3584 CS Utrecht, The Netherlands; 6 Department of Medical Oncology, Leiden University Medical Center, Albinusdreef 2, 2333 ZA Leiden, The Netherlands; a.j.gelderblom@lumc.nl; 7 Trial and Data Center, Princess Máxima Center for Pediatric Oncology, Heidelberglaan 25, 3584 CS Utrecht, The Netherlands

**Keywords:** osteosarcoma, clinical trial data, survival predictions, Cox model, machine learning, random survival forests, survival artificial neural networks

## Abstract

**Simple Summary:**

The medical field is increasingly interested in using machine learning (ML) to analyse data, sparking a debate on whether ML is better than traditional statistical modelling. The aim of our retrospective study was to investigate the use of statistical models versus ML to predict survival in patients with osteosarcoma using low-dimensional data from the EURAMOS-1 trial. Results showed that ML did not add much value in this context, which involved data from nearly 2000 patients and eight predictors. Traditional statistical models worked well without needing extensive complex training, unlike ML, and they showed better effectiveness overall because they are easy to understand and use. The article helps healthcare researchers assess different prediction models in similar low-dimensional contexts.

**Abstract:**

Since the mid-1980s, there has been little progress in improving survival of patients diagnosed with osteosarcoma. Survival prediction models play a key role in clinical decision-making, guiding healthcare professionals in tailoring treatment strategies based on individual patient risks. The increasing interest of the medical community in using machine learning (ML) for predicting survival has sparked an ongoing debate on the value of ML techniques versus more traditional statistical modelling (SM) approaches. This study investigates the use of SM versus ML methods in predicting overall survival (OS) using osteosarcoma data from the EURAMOS-1 clinical trial (NCT00134030). The well-established Cox proportional hazard model is compared to the extended Cox model that includes time-varying effects, and to the ML methods random survival forests and survival neural networks. The impact of eight variables on OS predictions is explored. Results are compared on different model performance metrics, variable importance, and patient-specific predictions. The article provides comprehensive insights to aid healthcare researchers in evaluating diverse survival prediction models for low-dimensional clinical data.

## 1. Introduction

Osteosarcoma is the most common primary bone cancer, primarily affecting children, adolescents, and young adults, with an annual incidence of three to four cases per million [1,2,3,4]. Since the mid-1980s, there has been little progress in improving survival of patients diagnosed with osteosarcoma [1,4,5]. Recent prospective clinical trials have achieved 5-year overall survival (OS) rates of 70–80% and about 45%, for patients with localized disease and known primary metastatic disease, respectively [3,6,7,8,9]. While predicting survival for this young population is of high importance, it poses significant challenges.

Survival prediction models play a key role in clinical decision-making, helping healthcare professionals in tailoring treatment strategies to individual patient risk. The increasing interest of the medical community in using machine learning (ML) to analyse medical data has sparked an ongoing debate on the value of ML techniques versus more traditional statistical modelling (SM) approaches. Statistical models can provide valuable insights into the influence of various predictors on the clinical outcome and generally yield easily interpretable results. ML methods, which require fewer assumptions about the data and the relationship between variables, promise enhanced predictive capabilities, yet introduce additional complexities in the implementation and have limited interpretability [10,11], which is extremely important for clinicians. Furthermore, ML techniques require precise operating conditions to perform well, including adequate data processing to ensure inputs facilitate effective learning and proper tuning of hyperparameters to prevent overfitting, which leads to results that do not generalise well to new data. As a consequence ML models become unsuitable for clinical prediction [12]. Selecting the appropriate methodology should be driven by a combination of factors, including software availability, the characteristics and complexity of the available data, the computational intensity of the analyses, and the level of skill required for successful model implementation [13,14]. ML methods are well-suited for analysing complex, heterogeneous, and high-dimensional data types generated in contemporary clinical settings [15]. Examples include medical imaging, physician-entered notes, sensor data, and various -omics data (such as genomic, proteomic, and transcriptomic). In such contexts, properly trained and implemented ML approaches, based on high-quality data, may provide additional insights and enhanced predictive capabilities. In other contexts, the use of ML can be considered excessive, introducing complications that may well be unnecessary, and its added value when dealing with low-dimensional clinical trial data is still debated.

In this study, a retrospective analysis of the EURAMOS-1 clinical trial data [16] is performed with the objective of comprehensively investigating and comparing ML and SM approaches in predicting the overall survival (OS) from time of surgery, in patients with osteosarcoma. Specifically, random survival forests (RSFs) [17,18] and survival neural networks (SNNs) [14,19,20] are compared to the simple Cox proportional hazard (PH) model [21] and an extended Cox model, in which time-varying effects are included for covariates that violate the PH assumption [22]. The Cox PH model is a well-established and widely used tool for survival prediction that relies on the PH assumption, which entails that each predictor has a constant multiplicative effect on the hazard function over time. A violation of the PH assumption implies that the effects of covariates change over time. For instance, a prognostic factor may have a stronger effect at the beginning of follow-up, which decreases over time. Such a time-varying effect can be modeled through extended Cox models. Additionally, complicated non-linear effects and high-order interactions between variables may be present, which can be easily captured by ML models such as RSFs and SNNs.

In each prediction model, eight predictors measured at surgery are considered. The results are compared in terms of different model performance metrics, relative variable importance, and OS predictions. Additionally, patients with specific disease profiles are employed to illustrate the outcomes of each prediction model. The aim of this article is to provide comprehensive insights that can help guide healthcare researchers in evaluating diverse survival prediction models in scenarios with low-dimensional clinical data.

## 2. Materials and Methods

### 2.1. Study Design

In 2001, the Children’s Oncology Group (COG), the Cooperative Osteosarcoma Study group (COSS), the European Osteosarcoma Intergroup (EOI), and the Scandinavian Sarcoma Group (SSG) established the European and American Osteosarcoma Studies (EURAMOS) collaboration, with the purpose of pooling resources and facilitating the study of osteosarcoma [23]. A total of 2260 patients aged 40 or younger with a newly diagnosed resectable osteosarcoma were recruited from 2005 to 2011 in the EURAMOS-1 clinical trial (NCT00134030) [16]. Patients received neadjuvant chemotherapy prior to resection of the primary tumor, and were subsequently classified in poor and good responders, according to the histological response as assessed in the resected specimen (poor: ≥10% viable tumor; good: <10% viable tumor). A subset of 1334 (59%) patients was randomised to treatment. Good responders received MAP (methotrexate, doxorubicin, cisplatin) or MAPinf (MAP plus ifosfamide and etoposide), while poor responders were allocated MAP or MAPIE (MAP by pegylated interferon). A detailed description of the trial and treatment protocol has been published previously [3,6,7].

### 2.2. Consort Diagram

**Figure 1:**
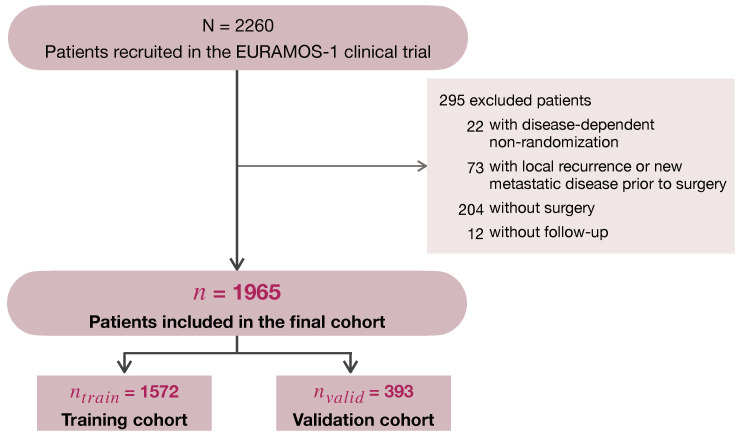
Consort diagram of patients included in the analysis.

As shown in Figure 1, among the 2260 patients recruited in the EURAMOS-1 trial, 295 (13%) were excluded due to one or more of the following criteria: (1) non-randomization due to progression of metastatic disease, the development of a new metastatic disease, or the presence of an unresectable disease; (2) a local recurrence or new metastatic disease recorded prior to the date of primary surgery; absence of (3) a surgery date or (4) follow-up. Both randomised and non-randomised patients were included in the analysis, as no benefit of experimental treatment was found in the primary analysis [6,7]. A subset of 1965 (87%) patients was found to be eligible for this analysis.

### 2.3. Survival Outcome and Relevant Predictors

The endpoint of interest was overall survival (OS), defined as the time to death from any cause since surgery (in years). Predictors of interest were selected based on clinical reasoning, considering (i) clinical input from Leiden University Medical Center (LUMC, The Netherlands), and (ii) the importance of particular prognostic factors for overall/event-free survival in existing medical literature on osteosarcoma. Adults, poor histological responders, the presence of metastases, and an axial tumor location have previously been found to be associated with decreased OS [3,6,7,24,25,26,27,28,29].

Eight predictors measured at surgery were selected: age (years), sex (male, female), tumor location (proximal femur/humerus, axial, other), absolute tumor volume (cm^3^), surgical excision as reported by the pathologist (wide/radical, marginal, intralesional/unknown), the presence of lung metastases (no, yes/possible), the presence of other metastases (no, yes/possible), and histological response (good: <10% viable tumor; poor: ≥10% viable tumor). Tumor location was defined by pooling the study variables “location” (proximal, diaphysis, distal, N/A not long bone) and “site” (femur, tibia, fibula, humerus, radius, ulna, scapula/clavicle, pelvis/sacrum, rib, spine, other), according to the definition used in previous analyses of the EURAMOS-1 trial [3,29].

### 2.4. Statistical Analysis

The reverse Kaplan–Meier method [30] was used to estimate median OS. The effects of the eight prognostic factors on OS predictions were evaluated using four different survival prediction methods: (A) Cox PH model [21], (B) extended Cox model including time-varying linear effects for predictors that violate the PH assumption [22], (C) RSFs [17], and  (D) SNNs modelled using partial logistic artificial neural networks [20] with a one-month interval partition of the follow-up period. The Cox models and the RSF utilized the exact time until the event occurred, measured since surgery. In contrast, in order to be able to perform the SNN analysis, the survival problem was converted into a classification problem where exact survival times were transformed into monthly time intervals denoting time since surgery [20].

As shown in Figure 1, the data were split into a training cohort (80%, $n_{\mathrm{train}}=1572$) and validation cohort (20%, $n_{\mathrm{valid}}=393$), while maintaining the event/censoring proportions of the original data. In both the training and validation cohorts, 20% of patients had missing values for one or more covariates. The highest percentage of missing data (17.7%) was observed for the prognostic factor absolute tumor volume. To make optimal use of the available data, missing values were imputed 10-fold in the training data, using the R-package mice (multivariate imputations by chained equations) [31]. The 10 imputation models used to impute the missing values in the training data were then applied to the validation data. This resulted in 10 imputed validation sets, each corresponding to one of the 10 imputed training sets.

The 10 training sets were used to first tune the RSF and SNN hyperparameters, and then estimate the Cox, RSF and SNN models. The 10 validation sets were used to evaluate model performance and to obtain the relevant OS predictions. The performance measures and OS predictions obtained for each validation set were subsequently averaged.

Statistical analyses were performed in the R-software environment (R version 4.3.1) [32], using the survival [33], randomForestSRC [18], and nnet [34] packages for the Cox, RSF, and SNN models, respectively.

#### 2.4.1. Hyperparameter Tuning of the ML Methods

Hyperparameter tuning of the ML methods was performed via grid search [35] to select the parametrization that achieves the best performance. This involved systematically searching through a range of possible parameter values to find the combination that optimized the performance. For the RSF, the hyperparameters to be tuned included: (i) number of tree estimators, (ii) maximum tree depth, (iii) minimum samples in a leaf, and (iv) maximum features for splitting. For SNN, the hyperparameters included the following: (i) number of nodes in the network hidden layer, and (ii) weight decay parameter value.

Model performance was evaluated in terms of concordance index (C-index) [36]. The C-index measures the discriminative ability of the model by calculating the proportion of observation pairs for which the model predictions and observed survival times are concordant. Values range from 0.5 to 1, with a C-index of 0.5 indicating a model with no discriminative ability and a C-index of 1 representing a perfect model. For the RSF, the C-index was computed using the ensemble mortality, which is the aggregated prediction of mortality risk made by the ensemble of decision trees that constitute the RSF model [17]. For the SNN, it was obtained using log-odds ratio of the predicted conditional hazard probabilities averaged across the time intervals. C-indices were first calculated for each training set using a 5-fold cross-validation approach, and then averaged over the 10 training sets to obtain a final mean cross-validated C-index.

#### 2.4.2. Model Estimation

For each training set, Cox and ML models were estimated including the eight previously described predictors. The coefficients estimated from Cox PH and extended Cox models from the 10 training sets were pooled using the Rubin’s rule [37].

The models were compared in terms of the rankings of predictor importance. For the Cox models, the effect of a predictor on the outcome was assessed by computing the relative predictor importance, using Heller’s relative explained risk [38]. For the SNN and RSF models, predictor importance was quantified using the connection weight method [39] and the variable importance measure [17,18], respectively. For each method, models were fitted to each of the 10 imputed datasets, with predictor importance first estimated separately for each model and then averaged. Finally, the methods were compared using the rankings of the averaged measures of predictor importance.

#### 2.4.3. Model Validation

Survival predictions were computed for each patient in the validation cohort. The performance of the models in predicting OS was assessed in terms of calibration and discrimination, two important measures used to assess the goodness of fit for statistical and ML predictive models. Specifically, the C-index [36], the time-dependent Brier score, and the time-dependent Kullback–Leibler (KL) divergence [40] were employed. For the Cox PH model, the C-index was computed from patients’ prognostic indexes, which are the sum of the regression coefficients multiplied by the value of their respective predictors. In the extended Cox model, time-dependent prognostic indexes were first obtained and subsequently averaged over time. Values for the time-dependent Brier score range from 0 to 1, with a lower score indicating better calibration of the model and accuracy of the predictions. A well-calibrated model ensures that differences between observed and predicted survival probabilities are small. The KL divergence is a non-negative value that measures the discrepancy between two distributions. A lower KL divergence indicates a closer match between the predicted and true survival distributions.

Calibration plots were employed to visualize how well the models’ predictions for the validation cohort align with the actual outcomes [41]. Data were divided into six equally sized prognostic groups, according to the 5-year OS predictions averaged over the models. For each model, the observed OS in each group, estimated using the Kaplan–Meier estimator [42], was plotted against predicted OS, at the time points of 3 and 5 years since surgery. Points that are scattered around the diagonal (x,y) indicate that the predicted probabilities match the observed probabilities well and that the model is well calibrated.

Finally, patients with specific disease profiles were employed to illustrate the outcomes of each survival prediction model. A visual comparison of the pattern and shape of the predicted survival curves was performed.

## 3. Results

### 3.1. Cohort Descriptives

Among the 1965 patients eligible for analysis, median follow-up time since surgery was 4.96 years (95% CI 4.87–5.09). At the end of study, 1499 (76.3%) patients were still alive, while 466 (23.7%) patients had died. Table 1 shows the characteristics at surgery for all patients, the 1572 patients in the training set, and the 393 patients in the validation set. Note that the proportions of dead and censored patients in both the training and validation cohorts were kept the same as in the original data.

### 3.2. Results from the Training Cohort

#### 3.2.1. Hyperparameter Tuning for ML Methods

To achieve optimal performance of the ML methods, the hyperparameters of both RSF and SNN were tuned using the grid search spaces shown in Table 2. Combining the grid of values for each hyperparameter, the grid search evaluated the performance of 10,108 RSFs and 50 SNNs. Table 3 shows the hyperparameter sets that achieved the top five performances in terms of the 5-fold cross-validated C-index, averaged over 10 training sets for both methods. In both cases, C-index estimates were similar for the top five combinations of tuning parameters. Configurations RSF-1 and SNN-1, which had the highest C-index, were used to fit the final RSF and SNN models on the training set.

#### 3.2.2. Variable Importance

Table 4 shows the hazard ratios (HRs) along with 95% confidence intervals (CIs), as estimated on the training cohort, for the simple Cox PH model and the extended Cox model with time-varying effects. All predictors had significant time-constant effects, except for age in the Cox PH model, and a marginal surgical excision (compared to a wide/radical surgical excision) in both models. Based on HR size, the prognostic risk factors with the strongest constant effects were a poor histological response, the presence of lung metastases and other metastases, and a proximal femur/humerus or axial tumor location. In the extended Cox model, histological response, age, and tumor localized in proximal femur/humerus had significant time-varying effects, modelled by an additional linear time-component in the coefficient. For these predictors, the time-varying hazard ratio can be calculated as $HR\left(t\right)=HR_{constant}\times HR_{time-var}^{t}$, where *t* is the time in years since surgery. These predictors violated the PH assumption, indicating that their effects were not fully captured by the time-constant terms in the simple Cox PH model.

**Figure 2:**
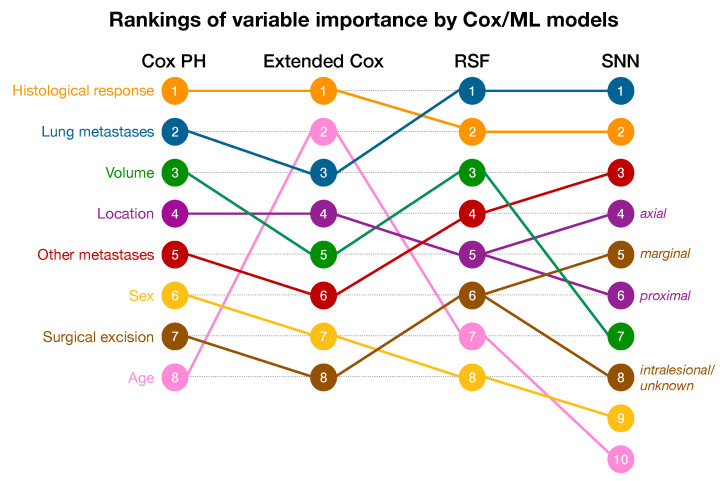
Rankings of variable importance for the Cox PH model, extended Cox model, random survival forest (RSF), and survival neural network (SNN).

Figure 2 displays the rankings of variable importance for the simple Cox PH model, extended Cox model, SNN and RSF. The rankings of the two Cox models were largely comparable, with different rankings for only two predictors. The simple Cox PH model identified tumor volume as the third strongest predictor, while the extended Cox model ranked volume fifth. More notable, age was identified as the second strongest predictor in the extended Cox model, while in the other three models, age was found to be one of the weakest predictors. The RSF and SNN models identified lung metastases as the most important predictor, followed by histological response, diverging from the Cox models, which prioritized histological response. The third most significant predictor was the presence of other metastases and tumor volume for the SNN and RSF models, respectively.

### 3.3. Results from the Validation Cohort

#### 3.3.1. Performance Measures and Goodness of Fit

Applying all fitted models to the validation cohort ($n_{\mathrm{valid}}=393$) yielded mean C-indices equal to 0.717 (range: 0.713–0.718), 0.719 (range: 0.716–0.721), 0.719 (range: 0.714–0.722) and 0.712 (range: 0.708–0.715) for simple Cox PH, extended Cox, RSF and SNN, respectively. These high values indicated a good discriminative ability of the models.

**Figure 3:**
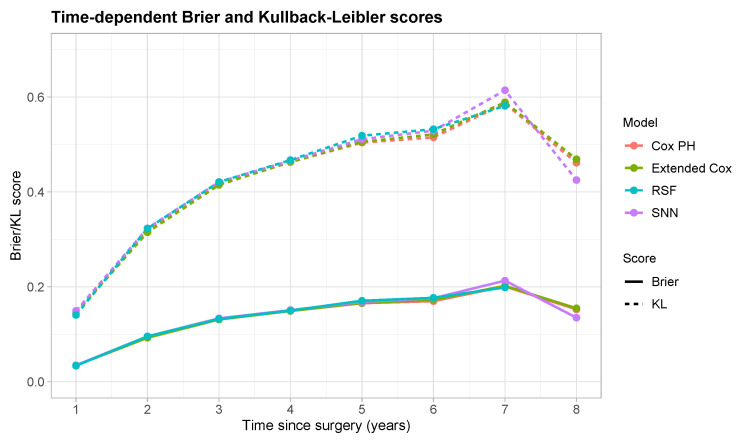
Time-dependent Brier (solid lines) and Kullback–Leibler (KL, dashed lines) scores. Lines are colored according to the different SM/ML models (pink: Cox PH model; green: extended Cox model; aquamarine: random survival forest, RSF; violet: survival neural network, SNN).

Figure 3 shows the time-dependent Brier (solid lines) and KL (dashed lines) scores, where lower values indicate better performance. For the RSF, no scores were calculated for times greater than 6.9 years after surgery due to the nature of the extrapolation procedure used to obtain predictions, which does not allow predictions to be made for times exceeding the latest observed event time in the training cohort (here, 6.9 years after surgery) [17]. Comparable performance was observed across all models, with low values of the Brier scores indicating that the models were well-calibrated. Low KL divergence values suggested a close match between predicted and true survival, especially in the first two years of follow-up.

**Figure 4:**
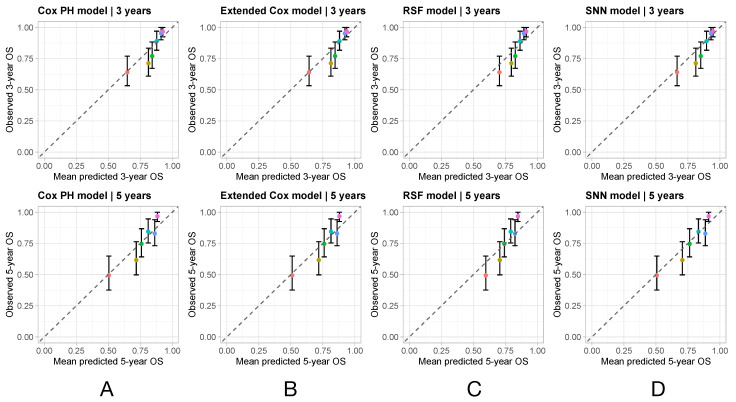
Calibration plots for overall survival (OS) at 3 years (**top panels**) and 5 years (**bottom panels**) since surgery. The observed OS obtained using Kaplan–Meier estimator is plotted against the mean predicted survival for patients in six equally sized risk groups identified by the different colors. Each column corresponds to a different model: (**A**) Cox PH model, (**B**) extended Cox model, (**C**) random survival forest (RSF), and (**D**) survival neural network (SNN).

Figure 4 shows, for each model, the calibration plots for OS at 3 years (top panels) and 5 years (lower panels) since surgery. In each panel, the points (representing risk groups) are scattered close to the diagonal, which is contained in the 95% CIs of the observed group survival, indicating that all four models were properly calibrated and produced predictions that corresponded well to actual outcomes.

#### 3.3.2. Predicted Survival Curves

**Figure 5:**
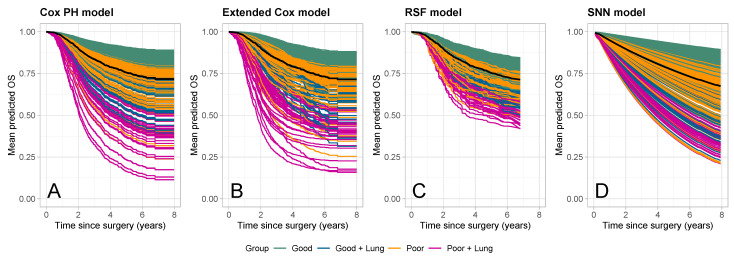
Predicted survival curves for all patients in the validation cohort ($n_{\mathrm{valid}}=393$). Curves are grouped and colored according to histological response and the presence of lung metastases (green: good histological response; blue: good histological response with lung metastases; orange: poor histological response; purple: poor histological response with lung metastases). Different panels correspond to the different prediction models: (**A**) Cox PH model, (**B**) extended Cox model, (**C**) random survival forest (RSF), and (**D**) survival neural network (SNN). The black line is the mean of the predicted curves.

Figure 5 displays the predicted survival curves for all patients in the validation cohort, grouped and colored according to histological response and the presence of lung metastases (green: good histological response; blue: good histological response with lung metastases; orange: poor histological response; purple: poor histological response with lung metastases). The order of the patients with respect to the groups was consistent across all models. Wide prediction spreads were observed for the Cox PH (Figure 5A), extended Cox (Figure 5B), and SNN (Figure 5D) models, while clustered predictions were observed for the RSF (Figure 5C). The black line represents the mean predicted survival curve averaged over the $n_{valid}=393$ patients in the validation cohort. These were comparable for the Cox PH, extended Cox, and RSF models, while lower values were observed for the SNN at later time points.

**Figure 6:**
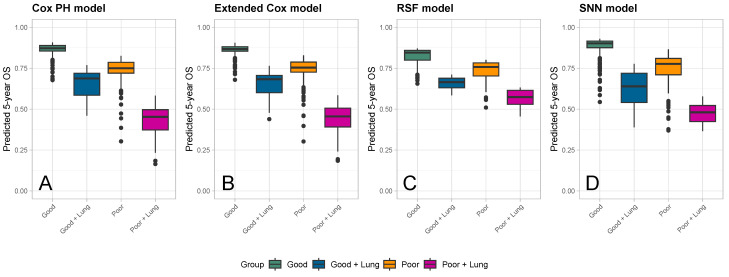
Boxplots of predicted 5-year OS probabilities for all patients in the validation cohort ($n_{\mathrm{valid}}=393$) grouped and colored according to histological response and presence of lung metastases (green: good histological response; blue: good histological response with lung metastases; orange: poor histological response; purple: poor histological response with lung metastases). Each panel corresponds to a different prediction model: (**A**) Cox PH model, (**B**) extended Cox model, (**C**) random survival forest (RSF), and (**D**) survival neural network (SNN).

Boxplots of the predicted OS probabilities at 5 years since surgery are shown in Figure 6, again grouped and colored according to histological response and the presence of lung metastases. The order of the boxplots with respect to the median values of each group was consistent across all models. Predicted values were similar for the Cox PH (Figure 6A) and extended Cox (Figure 6B) models. Compared to the Cox models, the 5-year OS probabilities predicted for patients with a good histological response and no lung metastases (green group) were lower for the RSF (Figure 6C) and higher for the SNN (Figure 6D). The 5-year OS probabilities predicted by the RSF for patients with a poor histological response and lung metastases (purple group) were found to be higher than in all three other models. Across all groups, the range of the 5-year OS probabilities predicted by the RSF was narrower than those from the two Cox models or the SNN.

### 3.4. Patient-Specific Survival Curves

**Figure 7:**
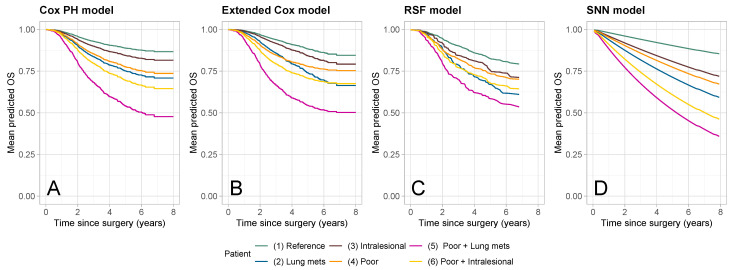
Patient-specific OS predictions for six patients with different specific traits (green: *reference*; blue: *lung mets*; brown: *intralesional*; orange: *poor hist*; purple: *poor hist + lung mets*; gold: *intralesional + poor hist*). Different panels refer to different prediction models: (**A**) Cox PH model, (**B**) extended Cox model, (**C**) random survival forest (RSF), and (**D**) survival neural network (SNN).

Figure 7 shows patient-specific predicted survival curves for six patients with the following traits:

1. *reference* (green): patient with reference values for all categorical predictors and mean values for the continuous predictors (see Table 1), i.e., a 15 year-old female patient with a good histological response, no lung metastases or other metastases, a radical/wide surgical exicison, and a tumor absolute volume equal to 200 cm^3^, located at any site except axial skeleton and proximal femur/humerus;
2. *lung mets* (blue): patient with reference/mean values for all predictors, but with the presence of lung metastases at surgery;
3. *intralesional* (brown): patient with reference/mean values for all predictors, but with an intralesional/unknown surgical excision;
4. *poor hist* (orange): patient with reference/mean values for all predictors, but with a poor histological response;
5. *lung mets + poor hist* (purple): patient with reference/mean values for all predictors, but with a poor histological response and lung metastases;
6. *intralesional + poor hist* (gold): patient with reference/mean values for all predictors, but with an intralesional/unknown surgical excision and a poor histological response.

Predictions were similar for up to 3 years after surgery for all four models, while the SNN had slightly lower values in the second half of the follow-up period (Figure 7D). Predicted curves for the SNN maintained the same ordering as in the Cox PH model (Figure 7A). In contrast, in the extended Cox model (Figure 7B) and the RSF (Figure 7C), the predicted curves for patients with *poor hist* (4, orange) and *intralesional + poor hist* (6, gold) intersected with the curve for the *lung mets* patient (3, blue). In the extended Cox model, this is due to the time-varying effect of a poor versus a good histological response. In the RSF, this may be due to interactions between histological response and other variables, which are not captured by the SNN. Predictions for the RSF were clustered, with under- and over-estimation of the survival probabilities for the *reference* (1, green) and *lung mets + poor hist* (5, purple) patients, respectively, when compared to the predictions from the Cox models.

## 4. Discussion

In response to the increasing interest in using ML methods in the medical field, this study investigated the application of ML versus Cox approaches to low-dimensional clinical data (nearly 2000 patients; eight predictors) from the EURAMOS-1 trial, for predicting OS since surgery in patients with osteosarcoma. Specifically, RSFs and SNNs were compared to the well-established Cox PH model and an extended Cox model, where a time-varying effect was included for predictors that violated the PH assumption. The models were compared utilizing variable importance measures, calibration plots, three performance measures (Harrel’s C-index, time-dependent Brier and Kullback–Leibler scores), and patient-specific predictions.

Similar performances were observed across the Cox and ML models, with comparable C-indices (slightly worse for the SNN) and nearly identical time-dependent Brier and KL scores, indicating good discriminative and calibration capabilities. This was confirmed by calibration plots, where points were scattered along the diagonal, indicating that the models were well calibrated. Overall, predictor importance was found to be comparable across models. Confirming previous literature [3,5,24,25,26,27,28,29], histological response and the presence of lung metastases were identified as the strongest predictors, ranking at the top in all Cox and ML models. Tumor volume, location and the presence of other metastases were found to be of middling importance, while surgical excision, sex, and age were found to be the least informative. The SNN assigned relatively less importance to tumour volume and more importance to the presence of other metastases. The extended Cox model assigned greater importance to predictors with a time-varying effect and was the only model that accorded high importance to the predictor age.

A limiting factor when comparing Cox models and ML techniques is the lack of a common metric in terms of interpretability. In Cox models, the effects of predictors on survival can be clearly defined and their effect sizes estimated, providing straightforward interpretation through HRs. In contrast, ML approaches are data-driven and do not assume predefined relationships between predictors. While this enables the capturing complex non-linear effects and interactions between variables, these relationships cannot be precisely quantified and it can be unclear whether a predictor’s effect on survival is positive or negative. This lack of interpretability of ML model results is particularly concerning in clinical applications.

Predicted OS curves for the validation cohort were found to be similar in range for the Cox models and the SNN, while they were clustered in the RSF, showing under- and over-estimation of the survival probabilities for high- and low-risk groups, respectively. Differences in predicted survival curves and their ordering between Cox PH and extended Cox models were attributed to the significant time-varying effects of histological response, age, and tumor location. These predictors violated the PH assumption, indicating that the simple Cox PH model incorrectly assumed their effects to be time-constant. The reasons for time-varying effects are complex and cannot be fully explained using the available data. For example, a proximal tumor could influence survival due to specific biological characteristics or overall patient health. Similarly, a poor histological response might impact survival through treatment resistance or aggressive tumor biology. Despite these considerations, the question remains complex and cannot be easily resolved. Patient-specific OS curves predicted by the SNN maintained the same ordering as the Cox PH model but had lower predicted values in the second half of the follow-up period. Due to this underestimation, SNNs may not be suitable for patient-specific predictions in this context. In contrast, curves predicted by RSF mirrored the ordering observed in the extended Cox model, suggesting effective capture of interactions between variables that the SNN did not achieve. However, these curves were clustered, suggesting that RSFs are unsuitable for predicting patient-specific survival.

A limiting factor in comparing RSF to other methods was the prediction time truncation at 6.9 years. In RSF, predictions are obtained by “dropping” a patient’s vector down the trees to one of the terminal nodes, which are informed by the event times observed in the training set [17,18]. This precludes extrapolation of predictions to unobserved time points. When interpreting SNN comparisons, it should be noted that the SNN method employed in this work, which uses partial logistic artificial neural networks, requires that the follow-up time be partitioned into intervals [20]. The effect of interval width has not been sufficiently investigated and merits consideration, as interval width and the resulting number of observations per interval will impact the variability of the point estimates, their interpretation, and the fit of the neural network itself.

In addition, ML methods heavily rely on well-conducted hyperparameter tuning to identify the best parameterization for a given dataset. However, finding a suitable combination of tuning parameters requires experience in training ML methods, making them disadvantageous for survival analysis in a simple clinical setting compared to conventional regression models for most non-specialist researchers. Moreover, even for expert researchers, the set of tuning parameters used in the final ML models may still be somewhat arbitrary. This is because the obtained performance under different parameterizations could be very similar, and choosing a different performance measure or specifying the performance measure differently is likely to result in a different set of optimal tuning parameter values, and hence, different predictions.

## 5. Conclusions

This is the first study in which ML techniques were applied to EURAMOS-1 clinical trial data and a comparison was made with traditional Cox models. The findings suggest that employing ML methods in a low-dimensional setting (involving nearly 2000 patients and eight predictors) is not worthwhile. Implementing the RSF and SNN required substantial time and resources without yielding significant advantages over the Cox models. The Cox models demonstrated satisfactory performance without the need for intensive model training, and are ultimately preferable for their straightforward interpretability and efficient implementation. In contrast, the implementation of ML methods necessitated careful consideration due to their extensive requirements in data pre-processing, hyperparameter tuning, and computational resources. Given these challenges and the loss of interpretability of predictor effects on OS, the use of ML approaches in a low-dimensional context should be primarily reserved as a supplementary tool to traditional statistical methods to explore model performance and highlight the advantages and disadvantages of each method. This approach ensures that the strengths of both ML and Cox methods are leveraged effectively without compromising implementation efficiency and interpretability in practical clinical settings.

## Figures and Tables

**Table 1:** Patient characteristics at surgery, along with survival outcome information.

	Training	Validation	Total
Cohort	$n_{\mathrm{train}}$ = 1572	$n_{\mathrm{valid}}$ = 393	n=1965
Age (in years).			
mean (s.d.)	15.1 (5.2)	15.8 (5.6)	15.3 (5.3)
min/max	4.3/40.7	5.3/40.5	4.3/40.7
Sex			
Female	646 (41.1%)	164 (41.7%)	810 (41.2%)
Male	926 (58.9%)	229 (58.3%)	1155 (58.8%)
Tumor location ^1^			
Other	1316 (83.7%)	325 (82.7%)	1641 (83.5%)
Axial	52 (3.3%)	12 (3.1%)	64 (3.3%)
Proximal femur/humerus	204 (13%)	56 (14.2%)	260 (13.2%)
Absolute tumor volume (cm^3^ × 0.54)			
mean (s.d.)	202.1 (268.6)	193.3 (242.7)	200.3 (263.6)
min/max	0.0052/2604	0.637/1870	0.0052/2604
Missing	278 (17.7%)	69 (17.6%)	347 (17.7%)
Surgical excision			
Radical/wide	1280 (81.4%)	326 (83%)	1606 (81.7%)
Marginal	185 (11.8%)	53 (13.5%)	238 (12.1%)
Other	107 (6.8%)	14 (3.6%)	121 (6.2%)
Lung metastases			
No	1266 (80.5%)	321 (81.7%)	1587 (80.8%)
Yes/Possible	306 (19.5%)	72 (18.3%)	378 (19.2%)
Other metastases			
No	1508 (95.9%)	381 (96.9%)	1889 (96.1%)
Yes/Possible	64 (4.1%)	12 (3.1%)	76 (3.9%)
Histological Response			
Good (<10% viable tumor)	792 (50.4%)	204 (51.9%)	996 (50.7%)
Poor (≥10% viable tumor)	734 (46.7%)	181 (46.1%)	915 (46.6%)
Missing	46 (2.9%)	8 (2%)	54 (2.7%)
Death status			
Censored	1199 (76.3%)	300 (76.3%)	1499 (76.3%)
Dead	373 (23.7%)	93 (23.7%)	466 (23.7%)
Follow-up time ^1^ (in years since surgery)			
median (95% CI)	5.01 (4.89–5.17)	4.87 (4.65–5.09)	4.96 (4.87–5.09)

^1^ Median follow-up time was estimated using the reverse Kaplan–Meier method [30].

**Table 2:** Grid search spaces for the hyperparameters of the Random Survival Forests (RSF) and Survival Neural Networks (SNN).

RSF Hyperparameters	
(i) number of tree estimators	100, 200, 500, 750
(ii) maximum tree depth	2, 3, 4, …, 18, 19, 20
(iii) minimum leaf samples	10, 20, 40, 60, …, 160, 180, 200
(iv) maximum splitting features	2, 3, 4, 5, 6, 7, 8
**SNN Hyperparameters**	
(i) number of nodes in the hidden layer	1, 2, 3, …, 8, 9, 10
(ii) weight decay parameter	0.0001, 0.001, 0.01, 0.05, 0.1

**Table 3:** Best model configuration for Random Survival Forest (RSF) and Survival Neural Network (SNN) from grid search.

	Random Survival Forests				
Top 5 Ranked Configurations	RSF-1	RSF-2	RSF-3	RSF-4	RSF-5
RSF hyperparameters					
(i) number of tree estimators	750	500	500	750	500
(ii) maximum tree depth	4	5	7	7	6
(iii) minimum leaf samples	60	60	80	60	80
(iv) maximum splitting features	2	2	2	2	2
Averaged cross-validated C-index ^1^	0.70964	0.70956	0.70955	0.70952	0.70951
	**Survival** **Neural Networks**				
**Top 5 Ranked Configurations**	**SNN-1**	**SNN-2**	**SNN-3**	**SNN-4**	**SNN-5**
SNN hyperparameters					
(i) number of nodes in the hidden layer	1	1	1	2	2
(ii) weight decay parameter	0.10	0.05	0.05	0.1	0.01
Averaged cross-validated C-index ^1^	0.70312	0.70277	0.70245	0.70192	0.70165

^1^ Averaged cross-validated C-index was obtained from 5-fold cross-validated C-indices averaged over the 10 imputed training sets.

**Table 4:** Cox PH model and extended Cox model estimated on training set ($n_{train}=1572$): hazard ratios (HRs) and 95% confidence intervals (CIs).

	Cox PH Model		Extended Cox Model	
	HR	95% CI	HR	95% CI
Age (years at surgery)				
Constant	0.994	0.974–1.015	0.942	0.908–0.977
Time-varying ^1^			1.022	1.010–1.034
Sex				
Female	1		1	
Male	1.264	1.018–1.570	1.244	1.028–1.504
Tumor location				
Other	1		1	
Axial	1.899	1.224–2.947	1.966	1.338–2.888
Proximal femur/humerus— Constant	1.389	1.051–1.837	2.756	1.700–4.468
Proximal femur/humerus—Time-varying ^1^			0.737	0.603–0.901
Absolute tumor volume (cm^3^)				
Constant effect	1.0007	1.0004–1.001	1.0007	1.0004–1.001
Surgical excision				
Wide/Radical	1		1	
Marginal	0.879	0.641–1.206	0.876	0.664–1.156
Intralesional/Unknown	1.434	1.002–2.052	1.386	1.010–1.900
Presence of lung metastases				
No	1		1	
Yes/Possible	2.422	1.945–3.015	2.435	2.009–2.952
Presence of other metastases				
No	1		1	
Yes/Possible	1.954	1.319–2.896	1.941	1.370–2.750
Histological Response				
Good	1		1	
Poor—Constant	2.147	1.719–2.682	4.777	3.125–7.303
Poor—Time-varying ^1^			0.729	0.632–0.841

^1^ In the extended Cox model, a time-varying effect (linear in the coefficient) was added to covariates that violated the proportionality assumption. For these variables, the time-varying hazard ratio is given by $HR\left(t\right)=HR_{constant}\times HR_{time-var}^{t}$, where *t* is the time in years since surgery.

## Data Availability

Data may be obtained from a third party and are not publicly available. A request to access the EURAMOS-1 trial data may be submitted to the MRC Clinical Trials Unit (CTU, London). The application requires completion of an analysis and data release request form, where the applicant provides a project summary (detailing the motivation of the data request, the background and objectives of their project, and the reasons for requesting this specific dataset), the data requirements (for this study, the anonymised individual-level data for all registered patients were requested, including (demographic) patient characteristics, disease characteristics, pathology and surgical information, treatment data, and major events), and details on the proposed publication, authorship and acknowledgements policy. Data applications are submitted to the Coordinating Data Center (CDC, London), and subject to review by the Trial Management Group and the Trial Steering Committee.

## Abbreviations

The following abbreviations are used in this manuscript:

CI	Confidence interval
EURAMOS	European and American Osteosarcoma Studies
KL	Kullback–Leibler score
HR	Hazard ratio
ML	Machine learning
PH	Proportional hazard
OS	Overall survival
RSF	Random survival forest
SM	Statistical modelling
SNN	Survival neural network
